# Beyond PlasticSurgeryGPT: The Imperative for Reasoning-Enhanced Large Language Models in Plastic Surgery

**DOI:** 10.1093/asj/sjaf122

**Published:** 2025-06-21

**Authors:** Partha Pratim Ray

The burgeoning integration of artificial intelligence (AI) into medical practice has yielded remarkable breakthroughs, yet the unique demands of surgical specialties continue to challenge generic large language models (LLMs). In their initial proof-of-concept study, Ozmen et al introduce PlasticSurgeryGPT—a GPT-2 model (OpenAI, San Francisco, CA) fine-tuned on 25,389 plastic surgery research abstracts spanning January 2010 to January 2024—to bridge this gap and elevate performance in clinical decision support, surgical education, and domain-specific research.^1^ By leveraging HuggingFace (Menlo Park, CA) and PyTorch (The Linux Foundation, San Francisco, CA) frameworks, the authors applied rigorous text cleaning and tokenization to construct a specialized corpus, then demonstrated measurable gains over the unmodified GPT-2 baseline across key language-generation metrics: BLEU (0.135519 vs 0.130179), METEOR (0.583554 vs 0.550498), and ROUGE-1 (0.216813 vs 0.215494). These improvements, although numerically modest, represent significant strides in capturing the semantic nuances of surgical discourse and affirm the promise of domain-tuned LLMs in plastic surgery.

Although PlasticSurgeryGPT marks a pivotal first step, the complexity of surgical reasoning extends far beyond pattern recognition in abstracts alone. Surgeons synthesize multimodal data—preoperative imaging, intraoperative findings, patient comorbidities, and postoperative outcomes—while navigating dynamic decision trees and weighing competing risks in real time. Current generative models remain tethered to static textual inputs and lack transparent reasoning mechanisms. To truly transform surgical practice, the next generation of plastic surgery–specific LLMs must integrate advanced reasoning architectures that support chain-of-thought explanations, retrieval-augmented evidence citation, and multimodal understanding.^2,3^

For example, reasoning-focused engines like OpenAI's o3 and DeepSeek R1 (High-Flyer, Hangzhou, China) enable chain-of-thought tracing, articulating stepwise clinical logic when recommending a flap selection or advising implant sizing. Embedding such capabilities within PlasticSurgeryGPT could allow a resident to query, “What considerations inform the choice between a latissimus dorsi flap and a DIEP flap for lower-extremity reconstruction?” and receive not only a concise recommendation but also the intermediate rationale—vascular anatomy, donor-site morbidity, patient athletic status—that underpins it. Similarly, Google’s (Mountain View, CA) Gemini 2.5 multimodal synthesis could fuse patient photographs, computed tomography angiograms, and histopathology reports with narrative summaries from surgical textbooks to generate context-rich operative plans. Anthropic's (San Francisco, CA) Claude 3.7 Sonnet, with its constitutional guardrails, can ensure that risk communication remains ethically grounded and free from stigmatizing language, a critical consideration when addressing body-image concerns and postoperative expectations.

Nevertheless, LLMs—even domain-tuned ones—are not infallible experts. They may overlook patient-specific nuances (for instance, an athlete's preference to avoid an abdominal donor site or a rhinoplasty patient's acceptance of a mild dorsal hump). Overreliance on AI for surgical or treatment planning without human oversight risks compromising individualized care. Moreover, potential medicolegal implications must be considered: if an AI-recommended option is declined by the surgeon or patient, or if a complication arises after following an AI suggestion, liability may become unclear. As the authors rightly note, rigorous validation of AI technologies is key. Partnering AI-driven analysis with expert physician review is likely superior to relying on AI alone, ensuring that model recommendations are vetted against clinical judgment and patient preferences.^4,5^

To build trust and safety, future studies should move beyond abstracts to incorporate full-text articles, case series, consensus guidelines, and large-scale outcome data. Embracing larger architectures—such as fine-tuning GPT-3.5 or LLaMA-based (Meta, Menlo Park, CA) networks—would provide the capacity to internalize complex anatomical terminology and rare complication profiles. Retrieval augmentation with explicit citations to peer-reviewed guidelines can guard against hallucinations, whereas software-as-a-medical-device frameworks will ensure compliance with regulatory standards, data privacy safeguards, and postmarket surveillance. Third-party audits and bias assessments are essential to verify equitable performance across diverse patient populations and surgical subgroups.

Thus, by advancing reasoning-enhanced, multimodal LLMs in concert with rigorous human validation and clear medicolegal frameworks, the plastic surgery community can harness AI not merely as an automated assistant but as a trustworthy collaborator—one that augments clinical expertise, enhances surgical education, and elevates patient care while maintaining the highest standards of safety and accountability.
